# Transmissibility studies of vacuolar changes in the rostral colliculus of pigs

**DOI:** 10.1186/1746-6148-5-35

**Published:** 2009-09-18

**Authors:** Timm Konold, John Spiropoulos, Melanie J Chaplin, Leigh Thorne, Yvonne I Spencer, Gerald AH Wells, Steve AC Hawkins

**Affiliations:** 1 Department of Pathology, Veterinary Laboratories Agency Weybridge, Woodham Lane, Addlestone, UK; 2 Royal Veterinary College, Infection and Immunity Research Group, North Mymms, Hatfield, UK; 3 Department of Molecular Pathogenesis and Genetics, Veterinary Laboratories Agency Weybridge, Woodham Lane, Addlestone, UK

## Abstract

**Background:**

Histopathological examinations of brains from healthy pigs have revealed localised vacuolar changes, predominantly in the rostral colliculus, that are similar to the neuropil vacuolation featured in the transmissible spongiform encephalopathies and have been described in pigs challenged parenterally with the agent causing bovine spongiform encephalopathy (BSE). Feedstuff containing BSE-contaminated meat and bone meal (MBM) may have been fed to pigs prior to the ban of mammalian MBM in feed of farmed livestock in the United Kingdom in 1996, but there is no evidence of the natural occurrence of a transmissible spongiform encephalopathy (TSE) in the domestic pig. Furthermore, experimental transmission of BSE to pigs by the oral route has been unsuccessful. A study was conducted to investigate whether the localised vacuolar changes in the porcine brain were associated with a transmissible aetiology and therefore biologically significant. Two groups of ten pigs were inoculated parenterally with vacuolated rostral colliculus from healthy pigs either born before 1996 or born after 1996. Controls included ten pigs similarly inoculated with rostral colliculus from New Zealand-derived pigs and nine pigs inoculated with a bovine BSE brain homogenate.

**Results:**

None of the pigs inoculated with rostral colliculus developed a TSE-like neurological disease up to five years post inoculation when the study was terminated, and disease-associated prion protein, PrP^d^, was not detected in the brains of these pigs. By contrast, eight of nine BSE-inoculated pigs developed neurological signs, two of which had detectable PrP^d ^by postmortem tests. No significant histopathological changes were detected to account for the clinical signs in the PrP^d^-negative, BSE-inoculated pigs.

**Conclusion:**

The findings in this study suggest that vacuolation in the porcine rostral colliculus is not caused by a transmissible agent and is probably a clinically insignificant change. The presence of neurological signs in pigs inoculated with BSE without detectable PrP^d ^raises the possibility that the BSE agent may produce a prion disease in pigs that remains undetected by the current postmortem tests.

## Background

Spongiform change within grey matter neuropil due to vacuolation of neuronal perikarya and neurites is one of the characteristic findings in the transmissible spongiform encephalopathies (TSE) of animals and man [1]. These slowly progressive neurological diseases include scrapie of sheep and goats, bovine spongiform encephalopathy (BSE) and Creutzfeldt-Jakob disease (CJD) of man. Close similarities in the biological properties of the infectious agents of BSE and the variant form of CJD (vCJD) in man, together with epidemiological evidence, has indicated the zoonotic potential of BSE [2]. The BSE epidemic in the United Kingdom (UK) has been recognised as feed-borne through the consumption of contaminated meat and bone meal (MBM) in commercial feed [3]. The inclusion of mammalian MBM in feed of any farmed livestock has been banned in the UK since 04 April 1996 [4], but non-bovine farm animals, including pigs, may have been exposed to contaminated feed prior to this date. Pigs are susceptible to parenteral challenge with the agent of BSE but oral challenge failed to produce disease [5]. In the course of these studies it was noted that saline inoculated control and otherwise unaffected challenged animals had some vacuolar changes in the brain, most notably in the superficial layers of the rostral colliculus [6]. The vacuoles were morphologically indistinguishable from those found at this site in BSE-affected pigs but were relatively few in number, restricted in extent and considered an incidental observation in normal pigs. Similar findings have been described in a survey for TSE in pigs in Ireland [7]. These authors examined the brains of the pigs immunohistochemically for disease-associated prion protein (PrP^d^), with negative results. Although the pig is not considered to be a species naturally susceptible to TSE, the occurrence of localised vacuolar changes in the brain raises questions as to the pathological significance of the change and a possible causal link to a latent endemic TSE in pigs.

This study aimed to investigate the potential biological significance of vacuolation in the rostral colliculus in porcine brains by parenteral challenge of pigs with an inoculum prepared from this neuroanatomical site. A successful transmission might also help to elucidate whether an endemic TSE-like agent may be responsible for the observed histopathological changes and, by using two temporally separated inocula sources, from pigs born before and after the MBM ban in 1996, whether the BSE agent was possibly implicated.

## Methods

All procedures involving animals were approved by the Home Office under the Animals (Scientific Procedures) Act 1986.

### Inocula preparation and inoculation

Piglets were allocated at random to the challenge groups.

#### Pre-1996 and post-1996 test groups

The rostral colliculi of 21 normal healthy culled sows, ten born prior to 1996 and eleven born after 1996, were dissected aseptically. Selection of these source groups was based on the premise that the pigs born prior to 1996 were fed a commercial ration, which may have contained MBM whereas pigs born after 1996 were fed a commercial ration in which the inclusion of MBM was prohibited in the UK. From each brain one colliculus was retained for histological examination and the contralateral colliculus was frozen. The frozen counterpart of those tissues that, on histological examination, presented with vacuolation were pooled to form the two inocula.

Two groups of ten 2-3 week-old commercial large white piglets (four female, six barrows in each group) were inoculated parenterally, each with one of the pooled inocula. Each piglet received a 10% homogenate of the brain material in sterile saline solution administered intracerebrally (0.5 ml), intravenously (1.5 ml) and intraperitoneally (8 ml) as described previously [5].

#### New Zealand control group (NZ group)

Brains of 13 cull sows from New Zealand (NZ), a country free from animal TSE, were sampled aseptically and the rostral colliculi pooled for inoculation. The brains of an additional five pigs from the same source were fixed in 10 per cent formol saline for subsequent histopathological examination of the rostral colliculus. Ten 2-3 week-old piglets (seven female, three barrows) were inoculated similarly to the test groups with the pooled rostral colliculus inoculum.

#### Positive control group (BSE group)

A pool of brainstems collected aseptically from two cattle affected by BSE and culled in 1999 was used for parenteral inoculation of a further ten piglets (five females, five barrows).

One barrow died on the day of inoculation; its replacement was an unchallenged female piglet, which was mixed with the BSE-challenged animals.

### Husbandry

Pigs of each group were housed separately throughout the study and had no nose-to-nose contact. Separate entrances for each group of pigs, together with dedicated protective clothing and animal husbandry equipment for each group was also maintained throughout the study. Initially, both challenged groups were housed in one building and shared the same air space whilst the NZ and BSE groups were housed in separate buildings attended by different animal husbandry staff. The pigs were kept in pens, each designed to hold two pigs, but some pigs were kept on their own to avoid aggression. Between 98 and 114 weeks post inoculation (wpi), pigs were moved to a single building, which had four separate pens with separate entrances for each challenged group but a common air space; all pigs were cared for by the same animal technicians. Pigs were reared and maintained throughout the study period on a specific pig ration, manufactured according to the Veterinary Laboratories Agency's own specifications, which was free from animal protein.

### Clinical monitoring

Pigs were monitored daily by animal husbandry staff. More detailed clinical monitoring commenced at between 33 and 52 wpi. Clinical examinations were usually conducted monthly and comprised assessments of behaviour (response to approach by a human), sensation/vision (response to probing of the neck with artery forceps and evaluation of the response to a threatening gesture towards the eye [menace response]) as well as locomotion. Clinical signs recorded in detail were over-reactivity to external stimuli or to approaching, such as flinching, grunting, squealing and/or moving away. Apprehension was defined as grunting, squealing or running away with vocalising when approached by the observing person. A prion disease was suspected when the following signs were displayed: a combination of apprehension and gait abnormalities (stiff or ataxic gait) or a combination of over-reactivity to external stimuli (menace response testing and response to neck prick) and gait abnormalities in the absence of other abnormalities that could have explained these signs. These criteria were based on the description of the signs of BSE in pigs in a previous study [5,8] and followed similar criteria as for the diagnosis of BSE in cattle where behavioural, sensory and locomotor changes are associated with BSE [9,10].

The animals were monitored for a maximum of five years after inoculation and then culled unless they displayed clinical signs suggestive of a TSE or any other intercurrent disease that required an earlier cull. The clinical assessments were initially carried out "blind" without knowledge of the inoculation status, when they were housed in separate buildings. Knowledge of the positive and negative control group was unavoidable after they were moved to the new accommodation although the identity of the unchallenged pig mixed with the positive controls remained unknown to the clinician.

The frequency of expressed clinical signs in pigs of each group prior to cull was compared by Fisher's exact test.

### Postmortem examinations

The brains of all pigs were examined histopathologically and immunohistochemically (IHC) and by Western immunoblotting (WB) for the presence of PrP^d ^or its proteinase-resistant form, PrP^res^. Brain sections subject to a neuropathological examination were the brainstem at the level of the obex and the rostral midbrain. In three pigs with neurological signs, additional sections comprising the rostral medulla, the caudal midbrain, cerebellum, the frontal and the parietal cerebrum were processed. Initially, the monoclonal antibody (mAb) used for IHC was mAb L42 (R-Biopharm AG, Darmstadt, Germany), which has been used successfully for detection of PrP^d ^in sheep and cattle [11]. This antibody had shown similar disease-specific immunolabelling in BSE-affected pig brain as the polyclonal mouse antiserum 1B3 used in an earlier experiment [6] but is consistently associated with relatively high levels of non-specific background immunolabelling (J Spiropoulos and GAH Wells, unpublished observation). Subsequently, mAb 2G11, (Institut Pourquier, Montpellier, France), which had been used successfully for the diagnosis of atypical scrapie of sheep [12], was evaluated and gave high levels of specific immunolabelling and negligible non-specific labelling in porcine BSE-affected brains and is now considered the antibody of choice for the identification of PrP^d ^in porcine samples (J Spiropoulos and YI Spencer, unpublished observation). This antibody was used in addition to L42 on brain sections of any pig testing positive by histopathological, WB or initial IHC examinations, on brain sections of an unchallenged control pig, and on brain sections of the three pigs that were subject to further neuropathological examination (see above).

Tissue sections were de-waxed and rehydrated routinely. Epitope demasking was performed by immersion of sections for 30 minutes in undiluted formic acid, then washed in running tap water for 15 minutes, followed by autoclaving at 121°C in citrate buffer pH 6.1 (8.8 mM tri-sodium citrate dihydrate, 1.3 mM citric acid in 2 litres purified water). Endogenous peroxidase was blocked using 3% hydrogen peroxide (100 volume) in methanol, and washing buffer used throughout the procedure was tris buffered saline, supplemented with 0.2% tween20 (TBST). Primary antibody was applied for one hour at room temperature (2G11 at dilution of 1/400 and L42 at 1/1000). Immunodetection used biotinylated goat anti mouse and avidin-biotin-peroxidase-complex (Vector Elite, Burlingame, USA) with diaminobenzidine chromogen prepared in McIlvane's citrate buffer. Sections were counterstained using Mayer's haematoxylin, then routinely dehydrated, cleared and mounted in dibutyl polystyrene xylene (DPX), before examination by light microscopy.

For the WB, fresh samples of caudal medulla of all pigs were subjected to the VLA Hybrid technique using PrP mAbs 6H4 (human aa 144-152 [13]), P4 (ovine aa 89-104 [14]) and an additional 'N' terminal mAb, 12B2 (bovine aa 97-115 [15], kindly provided by J Langeveld, CVI, Lelystad, Netherlands). The method is a modification of the commercial Prionics^®^-Check WB assay (Prionics AG, Zurich, Switzerland) and has previously been described as a test to distinguish scrapie from BSE [16]. For this study, selected porcine samples were duplicated with one set having the proteinase K (PK) treatment omitted in order to assess the binding capacity of the antibodies to the physiological form of PrP, porcine cellular PrP (PrP^c^).

Essentially, each sample was prepared by adding Prionics homogenisation buffer to give a 10% suspension. The homogenate was subjected to a clarifying centrifugation step for 5 minutes at 1,127 g at 10°C. The first aliquot of 100 μl was subjected to PK (100 μg/ml) treatment and incubated at 50°C for 45 minutes before being stopped with Prionics kit digestion stop. A second 100 μl aliquot was incubated without the addition of PK. Prionics sample buffer (100 μl) was added and heated to 105°C for 10 minutes. Triplicate gels were prepared by loading 10 μl of each denatured sample onto NuPAGE 12% bis-tris gels (Invitrogen, Carlsbad, USA) and electrophoresed for 45 minutes at 200 V.

Membranes were blocked with Prionics Blocking Buffer for 30 minutes and one was incubated with mAb 6H4 (included in the kit), one with mAb P4 (R-Biopharm, Darmstadt, Germany) and one with mAb 12B2. The immunoreactive PrP^res ^bands were visualised using an enhanced chemiluminescence system (CPD-Star, Tropix, Bedford, USA) and the signals were visualised using a Fluor S Multimager (Bio-Rad Laboratories Ltd., Hemel Hempstead, UK).

Additionally, the ligand-based EIA (IDEXX HerdChek BSE-Scrapie Antigen EIA, IDEXX Laboratories, Westbrook, USA), which does not use a PK digestion step, was performed on a limited number of samples. Approximately 0.1 g of thalamus (made to a 25% homogenate in deionised water) from one pig each from the pre- and post-1996 group and NZ-group and two pigs from the BSE group was examined without knowledge of the inoculation or TSE status.

Both conjugates provided for the test, one for cattle and one for small ruminants, were used separately on each sample. Samples were extracted and analysed according to the kit protocol. Negative cut-off values were 0.14 absorbance units for the bovine conjugate and 0.202 absorbance units for the small ruminant conjugate.

## Results

Table 1 lists the individual details, experimental outcome and pathological status of the pigs at the termination of the study.

**Table 1:** Individual animal details and pathological status of the pigs used in the study

**Animal identification**	**Inoculum**	**Sex**	**wpi**	**Experimental outcome**	**Postmortem result (HP/IHC/WB)**
PR459	None	F	N/A^a^	Intercurrent death (arthrosis)	-/-/-
PR471	Pre-1996	M	250	Intercurrent death (foot abscess)	-/-/-
PR486	Pre-1996	F	265	Killed at termination of study	-/-/-
PR484	Pre-1996	M	277	Intercurrent death (arthrosis & spondylosis)	-/-/-
PR470	Pre-1996	M	295	Killed at termination of study	-/-/-
PR472	Pre-1996	M	295	Killed at termination of study	-/-/-
PR475	Pre-1996	F	295	Killed at termination of study	-/-/-
PR478	Pre-1996	F	295	Killed at termination of study	-/-/-
PR482	Pre-1996	F	295	Killed at termination of study	-/-/-
PR467	Pre-1996	M	297	Killed at termination of study	-/-/-
PR469	Pre-1996	M	297	Killed at termination of study	-/-/-
PR477	Post-1996	F	209	Intercurrent death (cause undetermined)	-/-/-
PR480	Post-1996	M	250	Intercurrent death (tumour)	-/-/-
PR488	Post-1996	M	255	Intercurrent death (foot abscess)	-/-/-
PR468	Post-1996	M	295	Killed at termination of study	-/-/-
PR479	Post-1996	M	296	Killed at termination of study	-/-/-
PR481	Post-1996	M	296	Killed at termination of study	-/-/-
PR483	Post-1996	F	296	Killed at termination of study	-/-/-
PR487	Post-1996	F	296	Killed at termination of study	-/-/-
PR474	Post-1996	M	299	Killed at termination of study	-/-/-
PR476	Post-1996	F	299	Killed at termination of study	-/-/-
PR463	BSE	M	148	Recumbency	+/+/+
PR443	BSE	F	175	Recumbency	+/+/+
PR445	BSE	F	234	Arthrosis	-/-/-
PR442	BSE	F	294	Killed at termination of study	-/-/-^b^
PR441	BSE	M	294	Killed at termination of study	-/-/-
PR444	BSE	F	294	Killed at termination of study	-/-/-^b^
PR462	BSE	M	297	Killed at termination of study	-/-/-
PR460	BSE	F	297	Killed at termination of study	-/-/-
PR440	BSE	M	298	Killed at termination of study	-/-/-^b^
PR620	NZ-brain	F	17	Fracture of right femur	-/-/-
PR593	NZ-brain	F	84	Spinal abscess	-/-/-
PR596	NZ-brain	M	218	Arthritis	-/-/-
PR591	NZ-brain	F	275	Killed at termination of study	-/-/-
PR594	NZ-brain	F	275	Killed at termination of study	-/-/-
PR598	NZ-brain	M	275	Killed at termination of study	-/-/-
PR599	NZ-brain	F	275	Killed at termination of study	-/-/-
PR621	NZ-brain	F	276	Killed at termination of study	-/-/-
PR623	NZ-brain	F	276	Killed at termination of study	-/-/-
PR627	NZ-brain	M	276	Killed at termination of study	-/-/-

N/A Not applicable

HP Histopathology

F Female

M Male (castrated)

^a ^Culled at 231 weeks of age.

^b ^Neuropathological examination extended to representation of all brain regions.

### Clinical Findings

The frequency of certain clinical signs or combinations of signs according to experimental group at the last examination immediately prior to cull is displayed in Table 2. Statistical significant differences were only seen between the BSE-inoculated pigs and the other groups (*P *< 0.05).

**Table 2:** Presence of selected TSE-like clinical signs or combination of signs observed at the pre-cull assessment

**Clinical signs**	**Group A**	**Group B**	**Group C**	**Group D**	**A-B**	**B-C**	**B-D**
Repeatable over-reactivity to menace testing	2	6	2	0	ns	ns	0.0031
Apprehension towards observer	0	5	0	1	0.01	0.01	ns
Repeatable over-reactivity to probing of the neck	1	7	1	1	0.006	0.006	0.006
Stiff gait	0	4	3	0	0.033	ns	0.033
							
Ataxia	0	5	0	1	0.01	0.01	0.0498
							
Apprehension, over-reactivity & ataxia/stiff gait	0	5	0	0	0.0108	0.0108	0.0108
Apprehension & over-reactivity	0	5	0	0	0.0108	0.0108	0.0108
Apprehension & ataxia/stiff gait*	0	5	0	1	0.0108	0.0108	ns
Over-reactivity & ataxia/stiff gait*	0	8	0	0	0.0001	0.0001	0.0001

The numbers in the first four columns (groups A-D) refer to the number of animals displaying this particular sign or combination of signs. Columns 5-7 list the *P *value by group comparison; ns = not significant (*P *> 0.05).

Group A = NZ brain, includes pig that was not inoculated (N = 10)

Group B = BSE (N = 9)

Group C = Pre-1996 group (N = 10)

Group D = Post-1996 group (N = 10)

* Signs associated with a prion disease; the one animal in group D with multiple abscesses is listed for completeness although a prion disease was not suspected (see text).

Based on the presence of apprehension or over-reactivity to external stimuli and gait abnormalities, previously associated with experimental BSE in pigs, eight pigs of the BSE group, including the two pathologically confirmed cases, were considered as TSE suspects prior to cull.

### Pathologically confirmed BSE cases

#### PR463

At 143 wpi, this pig developed over-reactivity to tactile stimuli, which were consistent on subsequent examinations. It also developed a stiff gait at that time and was finally found recumbent with difficulty rising, which led to its cull at 148 wpi.

#### PR443

This pig developed over-reactivity to visual stimuli at 157 wpi, which continued to be observed on subsequent assessments, followed by stiffness of the gait. It eventually developed ataxia with hind limb weakness resulting in difficulty in rising (see additional file 1: PR443 BSE, which shows over-reactivity and ataxia). A whole body tremor was also present prior to cull at 175 wpi.

### BSE-inoculated pigs with no pathological evidence of prion disease

Generally, differences in the behaviour of pigs in the BSE-inoculated group compared to the other groups were observed after transport to new accommodation at 115 wpi; they appeared to be more nervous and over-reactive [see additional file 2: Pre-1996 group, additional file 3: post-1996 group and additional file 4: BSE group (unconfirmed), which shows the behavioural differences between the BSE-inoculated pigs and the pigs inoculated with porcine rostral colliculus].

This was most marked in two pigs, PR444 and PR442, which became very apprehensive and over-reactive, characterised by loud squealing and running away in apparent panic whenever approached or touched. This behaviour had not been seen in their previous accommodation. However, these two pigs appeared more inquisitive on occasions, when they would approach an observer with repeated short grunts or would follow an observer with repetitive grunting although any attempt to touch the animal would result in immediate withdrawal with squealing (see additional file 5: PR444 apprehension, which shows this behaviour). Eventually, these characteristic repeated short grunts were also elicited by merely entering the pen, and other pigs would join in [see additional file 4: BSE group (unconfirmed) with pigs grunting repeatedly]. Similar vocalising behaviour was also expressed by the unchallenged control pig kept with the BSE-challenged pigs - and to a much lesser degree - in some animals in the other three groups, although this did not coincide with the apparent panic expressed when the animals were approached.

Behaviour, such as squealing, grunting, ear flapping or running away when approached or when the response to external stimuli was tested, was more frequently and consistently displayed in the BSE-challenged animals compared to pigs in the other groups, with the exception of one animal, PR462, which displayed this behaviour only inconsistently.

Ataxia was observed in four BSE-challenged, pathologically unconfirmed pigs from between 218 and 281 wpi, including PR462 and PR442 (see additional file 6: PR442 ataxia). The latter was also over-reactive and apprehensive [see additional file 4: BSE group (unconfirmed), which shows this over-reactive pig (spray mark blue 5) at the end of the clip]. Although the other over-reactive animal, PR444, did not display ataxia, it had developed a stiff gait by 11 weeks prior to cull.

The combination of over-reactivity and ataxic or stiff gait was exhibited by eight BSE-challenged animals (Table 2; PR440, PR441, PR442, PR443, PR444, PR445, PR462 and PR463) which included the two pathologically confirmed cases.

Five animals of the BSE-challenged group displayed the combination of apprehension and an ataxic or stiff gait (PR440, PR441, PR442, PR444 and PR445). It was also displayed in one animal from the Post-1996 group, PR488. This pig presented with ataxia from 229 wpi, which coincided with the appearance of and treatment for an abscess on the dorsal neck. It continued to be uncoordinated when culled 26 weeks later due to a foot abscess; it became apprehensive, but was not over-reactive to external stimuli. As the display of apprehension coincided with the treatment, this sign was not considered to be associated with a prion disease. At postmortem examination multiple abscesses were also found in the abdomen. Histopathological examination of cervical, thoracic and lumbar spinal cord did not reveal any conclusive changes that could have accounted for the observed gait abnormality.

All five animals of the BSE-challenged group that displayed apprehension and an ataxic or stiff gait were also over-reactive to external stimuli.

PR460 was the only BSE-challenged pig not to display the combination of signs considered to be suggestive of a prion disease. This pig displayed over-reactivity to external stimuli prior to cull, which included both over-reactivity to the menace response testing and over-reactivity to neck probing. Over-reactivity to both of these tests was also displayed by PR440, PR442, PR444 (BSE-challenged group) and PR472 (Pre-1996 group).

PR593 (NZ group) became recumbent with paresis of the hind limbs, but was not ataxic prior to this event. Postmortem examination revealed abscessation involving the spinal cord.

Twenty-two pigs, both male and female, distributed across all groups (BSE group: three, NZ group: three, Pre-1996 group: eight, Post-1990 group: eight), presented with a tremor of the ears. This was either noticed on the first clinical assessment or developed over time and was most pronounced when pigs were stressed or excited, but was not evident in resting or sleeping pigs (see additional file 7: PR471 as an example of this tremor).

### Pathological Findings

#### Histopathology and Immunohistochemistry

Histopathological examination of the brains of test group animals and UK donor pigs revealed neuropil vacuolation of the rostral colliculus, which was also present in the brains of pigs sourced from NZ. This vacuolation resembled closely that described in healthy pigs of other studies [6,7]. Intraneuronal vacuoles were observed in brainstem sections at the level of the obex of all inoculated pigs, a feature which was also reported in these previous studies.

**Figure 1:**
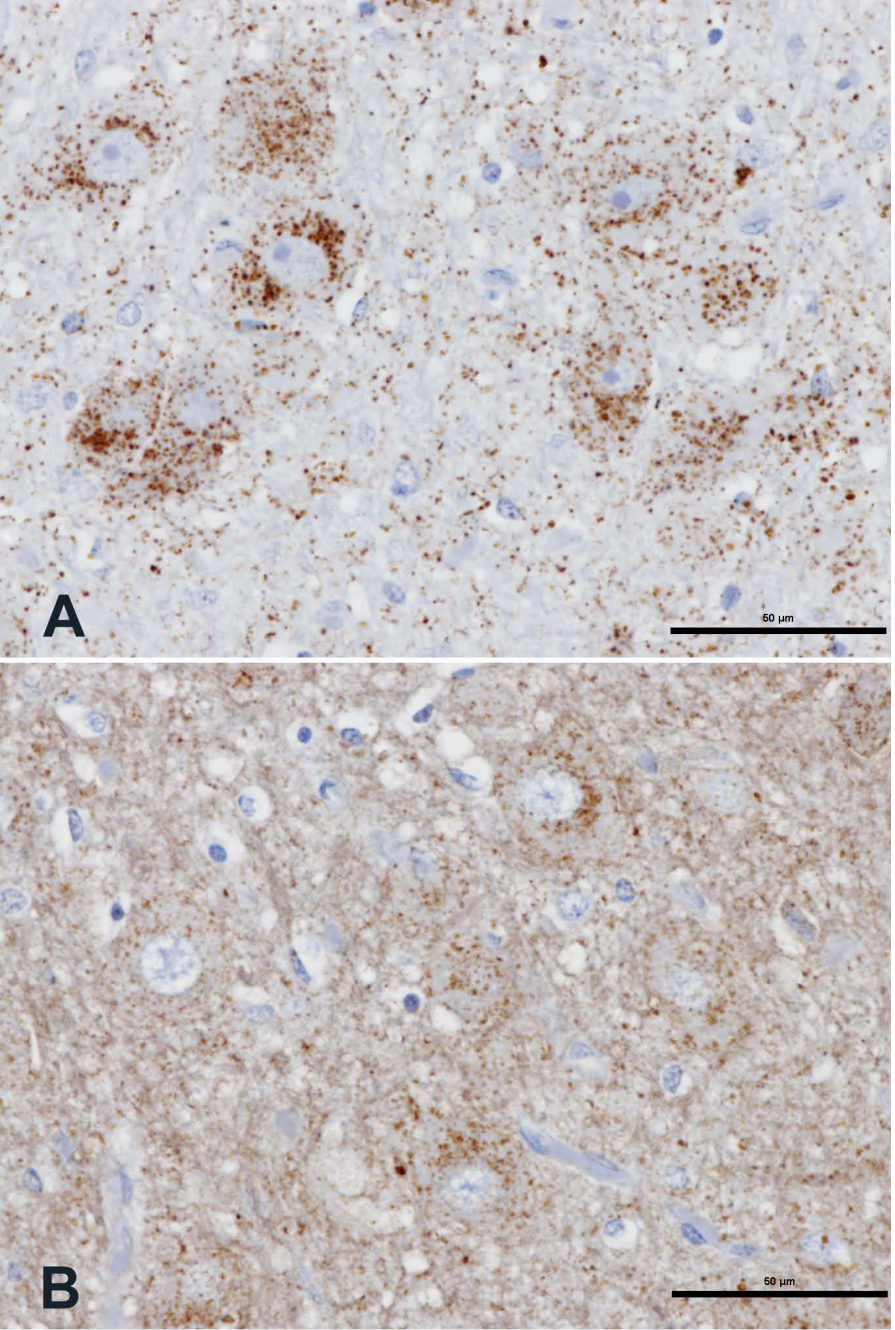
**Disease-specific immunolabelling in a BSE-inoculated pig**. Serial coronal sections (A and B) of rostral midbrain (periaqueductal grey matter) of pig PR443. A) Specific immunolabelling in neuronal perikarya and neuropil. Immunolabelling with mAb 2G11. B) Specific immunolabelling in neuronal perikarya and neuropil, masked to some extent compared to A) due to non-specific background immunolabelling with mAb L42. Scale bars: 50 μm.

PrP^d ^immunolabelling was found only in the brains of two pigs of the BSE-inoculated group, including the rostral colliculus. Such labelling was achieved with both mAbs L42 and 2G11, but specific labelling was most clearly evident with 2G11 (Figure 1A), unobscured by the non-specific background labelling experienced with L42 (Figure 1B). Intracellular (intraneuronal and intraglial) and neuropil-associated (fine granular and small aggregates) PrP^d ^immunolabelling was consistently observed with mAb 2G11 in these two pigs.

**Figure 2:**
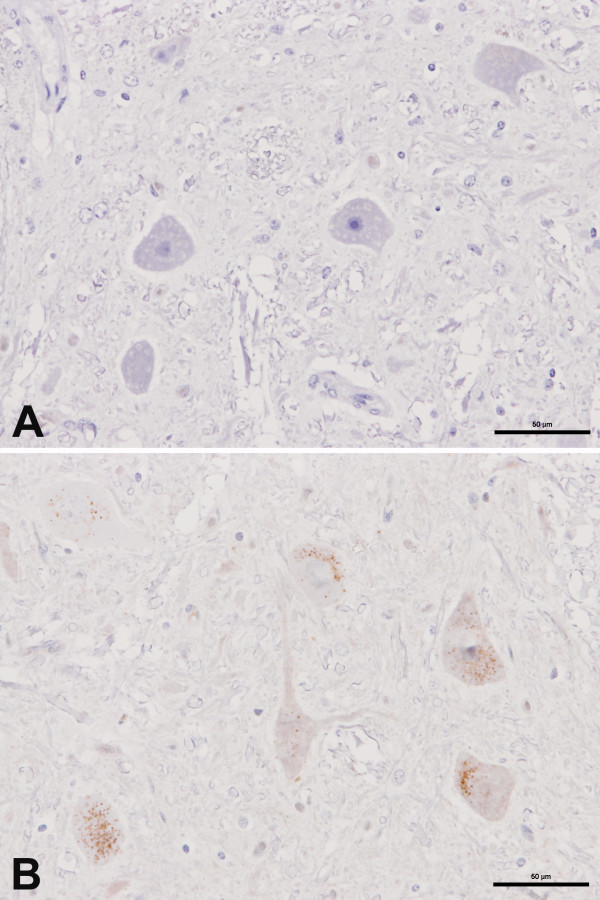
**Absence of disease-specific immunolabelling in an un-inoculated pig**. Serial coronal sections (A and B) of rostral midbrain (oculomotor nerve nucleus) of pig PR459. A) Absence of any labelling. Immunolabelling with mAb 2G11. B) Presence of disease-unspecific labelling of neuronal perikarya with mAb L42.

**Figure 3:**
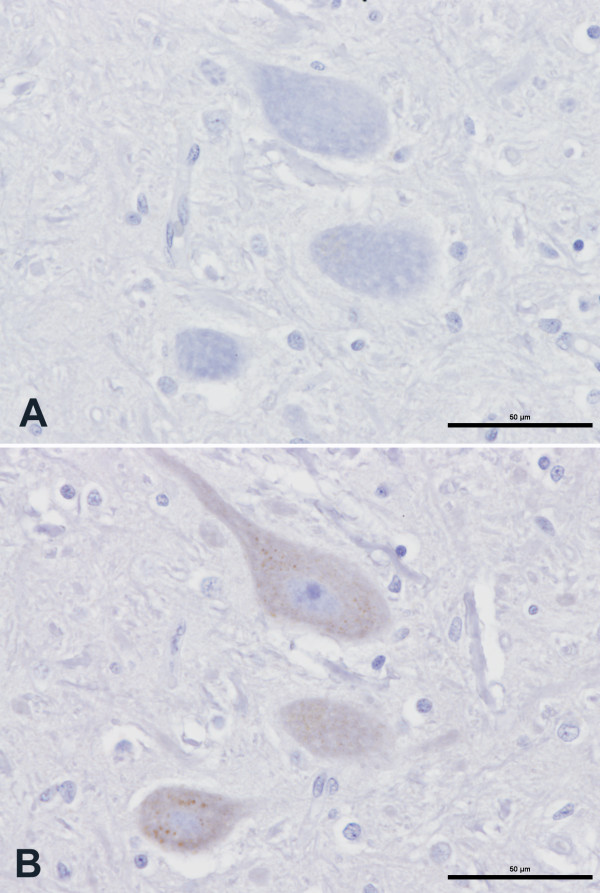
**Disease-unspecific, antibody-related immunolabelling in a BSE-inoculated pig presenting neurological signs in the absence of pathological evidence of disease**. Serial coronal sections (A and B) of rostral midbrain (oculomotor nerve nucleus) of pig PR442. A) No evidence of PrP^d ^immunolabelling with mAb 2G11. B) Presence of ubiquitous disease-unspecific labelling of neuronal perikarya experienced in pig brain with mAb L42. Scale bars: 50 μm.

No immunolabelling with mAb 2G11 occurred in brain sections, including the rostral colliculus, from an un-inoculated pig (Figure 2A) whereas immunolabelling of neuronal perikarya was evident with mAb L42 in the brain of this pig (Figure 2B). While no immunolabelling occurred with mAb 2G11 in the brain sections of the BSE-inoculated pigs that presented with neurological signs but were not confirmed to have prion disease pathology (Figure 3A), immunolabelling of neuronal perikarya was again evident with mAb L42 in the brains of these pigs (Figure 3B). This form of labelling with mAb L42 was considered to be immunologically specific but disease-unspecific labelling.

PrP^d ^immunolabelling using mAb 2G11 revealed variable fine filamentous labelling at certain brain locations (cingulate gyrus, PR440; ventro-lateral frontal cortex, PR444; hippocampus, PR442) of the three BSE-inoculated pigs in which further sections of brain were examined. Such labelling has been observed previously in sheep [17] and cattle (GAH Wells, unpublished observation) unaffected by TSE.

The brains of three pigs in the BSE group that were markedly apprehensive and displayed an ataxic or stiff gait (PR440, PR442, PR444), but which showed no evidence of a prion disease, had, in addition to mild or moderate neuropil vacuolation of the rostral colliculus, localised mild white matter vacuolation in the roof nuclei of the cerebellum (PR442) and in the thalamus (PR440, PR444), single vessels showing perivascular mononuclear cell infiltration (PR440, PR442, PR444) and focal mineralisation in the meninges (PR444). These changes were not considered to be clinically significant due to their limited extent and severity.

#### Western immunoblotting

**Figure 4:**
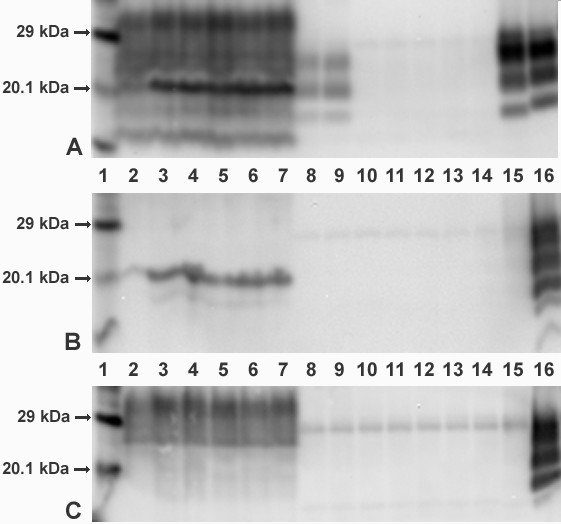
**Western immunoblot of caudal medulla of selected pigs from each group**. Lanes **1**      **Biotinylated molecular mass marker** *No PK*   *PK+*   *Animal ID* 2         8      BSE group (positive) - PR463 3         9      BSE group (positive) - PR443 4         11     Pre-1996 group (negative) - PR469 5         12     Post-1996 group (negative) - PR476 6         13     BSE group (negative, clinical suspect) - PR442 7         14     Un-inoculated pig (negative) - PR459 10     NZ-brain (negative) - PR591 15     Bovine BSE positive control 16     Ovine scrapie positive control VLA Hybrid technique (discriminatory WB) using core mAb, 6H4 (Figure A), and 'N' terminal mAbs, P4 (Figure B) and 12B2 (Figure C), to distinguish scrapie from BSE. Treatment with and without PK was used to visualise PrP^res ^and PrP^c ^respectively.

PrP^res ^was detected with mAb 6H4 by WB in the brain extracts of the two pigs in the BSE group (lanes 8-9, Figure 4A), but not with mAbs P4 (lanes 8-9, Figure 4B) or 12B2 (lanes 8-9, Figure 4C); PrP^d ^was detected by IHC in both pigs. PrP^res ^was not detectable with any of the antibodies in the brain samples of the other pigs (lanes 10-14, Figures 4A, B and 4C). When brain samples were not treated with PK, characteristic protein bands representing porcine PrP^c ^were obtained in all tested pigs regardless of post-mortem TSE status using mAb 6H4 and mAb 12B2 (lanes 2-7, Figure 4A and 4C), but not with mAb P4 (lanes 2-7, Figure 4B).

#### EIA test

The results confirmed those obtained by WB and IHC examinations (Table 3).

**Table 3:** EIA result on thalamic samples of selected pigs

**Animal (inoculum)**	**Small ruminant conjugate** **(OD at 450-620 nm)**	**Bovine conjugate** **(OD at 450-620 nm)**	**Test result**
PR463 (BSE)	2.86	2.926	Positive
PR442 (BSE)	0.03	0.029	Negative
PR469 (Pre-1996)	0.03	0.032	Negative
PR476 (Post-1996)	0.022	0.022	Negative
PR591 (NZ brain)	0.032	0.027	Negative

OD Optical density

## Discussion

This study aimed to investigate the possible biological significance of vacuolar changes observed in the porcine rostral colliculus. Hypothetically, this vacuolation, resembling closely the spongiform change featured in TSE, might represent an endemic, unprecedented, localised form of TSE in this species. With the apparent ubiquitous occurrence of the change in domesticated pigs, this scenario, in the absence of a species barrier, might seem more likely than a high prevalence primary transmission of the BSE agent from cattle assuming similar titres of infectivity. The latter is especially unlikely given the previous failure of oral transmission of BSE to pigs [5]. Clearly, however, titre could be critical in effecting such a transmission. While this seems unlikely in the absence of any pathological evidence of transmission from vacuolated rostral colliculus in this study, a transmissible agent cannot be entirely excluded as the cause of the lesion. This uncertainty is inevitably compounded in large domestic animal experiments where recipient group size is restricted on cost and in long duration studies often further reduced by incidental and age-related illnesses. The low proportion of pathologically confirmed BSE cases in the positive control group presents further difficulty in interpretation of the findings since it suggests that if the probability of detection of prion disease in the test groups was similar or less, a negative result was the more likely outcome.

Vacuolation of the rostral colliculi was a feature of all of the brains studied and there was no significant difference in the (mildly expressed) clinical signs between pigs inoculated with rostral colliculi from different sources. The rostral colliculus is involved in coordinating eyeball movements, pupil constriction and head and neck control [18]. Lesions in this structure have been associated with altered responses to visual stimuli and deficits in visual perception and orientation [19,20]. Although we did not specifically assess pigs for their visual perception and orientation skills, no apparent visual impairment was detectable based on the evaluation of the menace response and the behaviour of the pigs in their environment. This vacuolar change appears therefore to be a non-specific pathological finding in pigs, as concluded previously [6] and as has been observed in cattle and other species [1,21].

Tremor was a neurological sign observed in pigs of all groups. Congenital tremor, related to developmental myelinopathies, has been well described in pigs and may be hereditary (e.g. in the Landrace breed) or caused by infections [22,23]. It is a generalised tremor involving the limbs, the body and the head [24] and, in its commonest form, usually disappears with increasing age in surviving piglets. This type of tremor, which ceases at rest and may be termed "action-related repetitive myoclonus" [25] or "essential tremor" [26], has also been observed in dogs and humans without evident cause [26,27]. It has also been described in association with neurotoxins and autoimmune disorders [25] and does not appear to represent the intention tremor seen in cerebellar disease due to the absence of other neurological signs of cerebellar dysfunction, in particular dysmetria or an impaired menace response. The tremor seen in the pigs of this study was generally confined to the head and affected predominantly the ears, with the exception of the confirmed BSE case PR443, which exhibited a body tremor. Tremor in the shoulder regions, flanks and of the ears has also been observed in pigs experimentally affected with BSE [8]. In our study, not all pigs in one litter were affected but occurrence in pigs of all groups is not suggestive of an association with a spongiform encephalopathy. That the tremor, at least in some cases, appeared to develop over time, is suggestive of an acquired rather than a congenital disorder.

Intracranial injection may occasionally cause neurological abnormalities, such as medial strabismus and exophthalmos in cattle inoculated intracerebrally (T Konold, unpublished observation) but no such signs were detected in the pigs in this study, and histopathological examinations of the brains (caudal brainstem and the rostral midbrain in all and additional brain regions in one pig [PR440]) of those pigs affected by tremor did not reveal significant changes.

The clinical signs in the BSE-inoculated pigs (positive control group) included signs of a systemic neurological disorder in addition to tremor. Only two of nine pigs inoculated with BSE developed a prion disease that was confirmed by postmortem tests. The apparent poor attack rate in this group may be explained by a combination of the species barrier effect and possible low titre of the BSE inoculum as it is well documented in wild-type mice [28]. The inoculum was not titrated in mice to determine the infectious titre but both BSE-positive pigs were culled with clinical signs at 148 and 175 wpi compared to a range of 74 to 163 wpi in a previous study [5]. Although, in this previous study, the pooled inoculum (made from four BSE-affected cattle brainstems [BSE1-4]) was also not titrated, transmission studies in mice indicate that the tissue that contributed to the pool had a titre of approximately 10^5 ^intracerebral (i.c.) ID_50 _mouse infectious units per g [5,29]. Furthermore, the transmission rate in the pigs was high (eight of eight pigs surviving beyond 50 wpi presented with a prion disease clinically and/or pathologically). While the cull times in the present study might therefore suggest that the infectious titre of the inoculum was probably slightly lower than for the previous study, this does not provide a satisfactory explanation for the low attack rate given the optimal parenteral routes of inoculation used in both studies.

WB examination of brain samples with or without PK treatment revealed that the core mAb 6H4 is able to detect porcine PrP^c ^and PrP^res^, which suggests that this test should be suitable for postmortem diagnosis of TSEs in pigs. The predominance of the monoglycosylated band in the positive pig samples compared to the bovine BSE control where the diglycosylated band predominates is a phenomenon that has been reported previously on passage of classical BSE to transgenic mice expressing porcine prion protein (poTg mice) and may be associated with the PrP structure in this species [30].

The 'N' terminal mAb P4, which is used successfully to discriminate between scrapie and BSE in sheep and cattle [31,32], does not appear to be suitable for use in porcine species. However, mAb 12B2 shows detection of PrP^c ^but not PrP^res ^in the porcine samples and is therefore a suitable alternative. Similarly, the BSE-Scrapie Antigen EIA, which may detect potentially proteinase-sensitive isoforms of PrP^d ^although it has not been validated for use in porcine tissues, was able to diagnose the BSE-inoculated pig that was positive by the other postmortem tests whereas a negative test result with similar absorbance units was obtained for all other pigs, including a BSE-inoculated pig with neurological signs, which were negative by other tests. We did not determine the genotype of the pigs, but based on previous reports on the relative homogeneity of the porcine PrP gene [33-35] it is unlikely that the genotype of the pigs affected the diagnostic sensitivity of the postmortem tests.

Eight of the nine pigs in the positive control group, surviving at the time of termination of the study (at approximately 290 wpi), were considered to be affected by a neurological syndrome. BSE was suspected in these pigs because the signs were similar to those seen in the pathologically confirmed pigs culled at 148 and 175 wpi and to those reported in previous studies [5,36]. For example, pigs with BSE in a previous study became easily frightened or persistently approached attendants with continual vigorous vocalisations [8], which was similarly observed in the BSE group in this study (see additional file 5: PR444 apprehension as an example of the repetitive vocalisations). The presence of behavioural or sensory changes in combination with an abnormal gait is frequently found in BSE-affected cattle, and this finding in the BSE-challenged pigs in the absence of any other disease of pigs that could have explained these signs was suggestive of a prion disease. We are not aware of any known single nervous disease entity of adult pigs described in the veterinary literature that could present with the combination of signs described, without marked progression over several months and without obvious histopathological changes.

The clinical protocol used for this study differed slightly from the protocol used in previous pig studies (no previous testing of the response to external stimuli) because it was adapted from the assessment of cattle for signs of BSE, grouped into behavioural, sensory and gait changes [10]. The various vocalisations of pigs were particularly assessed since vocal responses have been frequently used to study behaviour and stress in pigs [37,38]. Squealing is associated with increased stress or pain in pigs [39-41] and was in this study interpreted as increased over-reactivity (when in response to external stimuli) or apprehension (when in response to an observer's approach). Short rapidly-repeated grunts as produced by pigs in the BSE groups - and to a lesser degree by pigs in the other groups - appear to have a greeting or threat function [37]. Aggressiveness was however not observed in these pigs; in fact, the pigs would run away when approached. These vocalisations occurred later in the incubation period and - in combination with the squeals and grunts after stimulation of the animals - were interpreted as behavioural changes.

The combination of behavioural, sensory and gait changes, seen in five of these pigs, was suggestive of a diffuse brain disease [42]; behavioural or sensory changes observed in the other pigs of this group without clear neurogenic gait deficits may have been caused by a disease not affecting the central nervous system or, under certain conditions, may be part of normal pig behaviour. The histopathological changes in the brains of the pigs with the most severe clinical signs were either considered a "normal" feature of adult and sub-adult pigs [5-7] or regarded as incidental changes of no clinical significance. We were therefore unable to determine the cause for the observed neurological signs although the presence of these signs in BSE-infected pigs culled at termination of the study, which were not observed similarly in the other groups, raises the possibility that these animals may have developed a prion disease that did not present with pathological changes and remained undetected by the current statutory diagnostic tests. Two factors that could have resulted in different behavioural responses between the BSE-inoculated pigs and pigs of the other groups were considered: handling of groups by different husbandry staff prior to transport of all pigs to one building, which may have affected their subsequent behaviour, and - at the same time - mixing of pigs of each group, which were previously kept in groups of two. Both factors have been investigated in porcine behaviour studies [43-46] with sometimes conflicting results. However, even if these factors were relevant, it would not explain why the behaviour of all pigs was unremarkable prior to transport regardless of group whereas after transport, when all pigs were subjected to the same 'treatment' (mixing and handling by the same person), only pigs in the BSE-inoculated group developed behavioural changes. In fact, stress, such as transport, is known to trigger signs of BSE in cattle [47] and may have had the same effect on pigs challenged with BSE.

A transmission study of naturally occurring BSE in poTg mice failed to induce disease or detectable PrP^res ^accumulation in mice on first passage but subsequent passage of brain homogenates from these mice in poTg mice produced a prion disease with clinical signs [30]. The authors concluded that there was a strong species barrier between cattle and pigs and that exposure to bovine prions may lead to subclinical infection. This is in contrast to our findings where pigs were clinically affected yet had no evidence of prion disease pathology. Further transmission of brains from clinically affected pigs to pigs or poTg mice to overcome the species barrier would be necessary to determine whether these pigs were indeed affected by a prion disease. Clinical signs of a prion disease have been observed in mice infected with prions in the absence of detectable prion protein [48,49]. If the same applied to transmission of BSE to pigs, any survey to investigate the presence of BSE in the pig population based on conventional diagnostic methods, might not reveal the true status of the population. In fact, surveys in several European Countries have all been based on PrP^d ^detection methods and found no evidence of a TSE in pigs [7,50,51].

The UK Food Standard Agency (FSA) considered the risk associated with feeding of MBM in its review of BSE controls in 2000 [52] and suggested that recycling within a species should be banned completely. Although there were suggestions to allow feeding of pig-derived MBM to poultry based on the assumption that pigs do not carry TSE, it was noted in agreement with the UK Spongiform Encephalopathy Advisory Committee (SEAC) that "if infectivity were to be present in pig MBM fed to chickens it would not be inactivated in the chicken intestine. If infected chicken tissues were then incorporated into pig feed it would amount to intra-species recycling." The FSA also raised concerns about the risk of assuring that feed streams for different species (e.g. pigs and cattle) were adequately separated. Contamination of feed for ruminants with traces of BSE-contaminated food from other species has indeed been proposed as the main reason for the occurrence of bovine BSE cases born after the MBM ban [53,54]. The issue of feeding pig-derived MBM to poultry and vice-versa has again been raised recently with the European Food Safety Authority (EFSA) as a request from the European Parliament for an opinion on the BSE related public health risks of certain animal proteins in animal feed [55]. It was concluded that given current control measures risks to public health would be negligible because BSE has not been identified in pigs or poultry under natural conditions. However, if a carrier status in pigs existed that was able to transmit disease but did not present with detectable PrP^d ^it would argue strongly against feeding of non-ruminant MBM to non-ruminants.

## Conclusion

The findings suggest that vacuolation in the rostral colliculus is a common feature of porcine brains without causing evident clinical signs and does not represent a localised form of a transmissible spongiform encephalopathy. The presence of neurological signs in pigs challenged with BSE in the absence of detectable disease-associated prion protein or other visible pathological changes raises the possibility that the BSE agent may cause a chronic disease that remains undetected by current prion disease phenotypic definitions and postmortem tests.

## Authors' contributions

TK carried out the clinical assessments and drafted the manuscript. JS and GAHW conducted the neuropathological investigations and helped to draft the manuscript. MJC was responsible for the Western immunoblot technique, LT for the EIA and YIS for the optimisation of the immunohistochemical technique. SACH managed and coordinated the study. All authors contributed to the drafting of this manuscript and read and approved the final manuscript.

## Supplementary Material

Additional file 1 **PR443 BSE**. This BSE-inoculated pig (BSE confirmed by postmortem tests) displays over-reactivity to visual stimuli (head flinch and moving away at menace response testing) and hind limb ataxia at 175 wpi. Click here for file

Additional file 2 **Pre-1996 group**. General behaviour of the group inoculated with brains from pigs culled prior to 1996, at 272 wpi. The pigs within this group are inquisitive and do not react to probing of the neck or menace response testing, even when lying down, similar to the Post-1996 group. Click here for file

Additional file 3 **Post-1996 group**. General behaviour of the group inoculated with brains from pigs culled after 1996, at 272 wpi. There is no obvious over-reactivity to tactile (probing of the neck) or visual stimuli (menace response testing) in standing or lying pigs within this group, similar to the Pre-1996 group. Click here for file

Additional file 4 **BSE group unconfirmed**. General behaviour of the BSE-inoculated group at 274 wpi, at which time both BSE-positive pigs had been culled. The pigs in this group appear to be more vocal. Note the repetitive grunting of the pigs when the pen is entered. The pigs display over-reactivity to tactile and visual stimuli and apprehension, characterised by vocalising, ear flapping and running away from the assessor. Click here for file

Additional file 5 **PR444 apprehension**. This BSE-inoculated pig (BSE not confirmed by postmortem tests) approaches the observer with repetitive grunting but displays apprehension, characterised by running away with vocalising, when approached by the observer. This behaviour had been expressed after transport to new accommodation at 116 wpi, which made testing for visual and tactile over-reactivity impossible. Click here for file

Additional file 6 **PR442 ataxia**. Of the three pigs leaving the pen, the second (PR442, spray mark Blue 5) displays an abnormal gait with abnormal swaying. When observed individually re-entering the pen, this BSE-inoculated pig (BSE not confirmed by postmortem tests) exhibits hind limb ataxia with occasional, audible dragging of the hind claws. This was a consistent finding from 228 wpi. Click here for file

Additional file 7 **PR471 ear tremor**. A marked ear tremor is present in this pig whilst exploring an observer or whilst drinking. This tremor disappears when the animal is at rest. Tremor in this pig was first noticed at 106 wpi. Click here for file
